# Effects of Rifaximin on Luminal and Wall-Adhered Gut Commensal Microbiota in Mice

**DOI:** 10.3390/ijms22020500

**Published:** 2021-01-06

**Authors:** Marina Ferrer, Mònica Aguilera, Vicente Martinez

**Affiliations:** 1 Department of Cell Biology, Physiology and Immunology, Universitat Autònoma de Barcelona, 08193 Barcelona, Spain; marinaferrerclotas@gmail.com (M.F.); monica.aguilerap@gmail.com (M.A.); 2 Neuroscience Institute, Universitat Autònoma de Barcelona, 08193 Barcelona, Spain; 3 Centro de Investigación Biomédicaen Red de Enfermedades Hepáticas y Digestivas (CIBERehd), Instituto de Salud Carlos III, 28029 Madrid, Spain

**Keywords:** dysbiosis, gut commensal microbiota, host-bacterial interaction systems, immune markers, rifaximin, toll-like receptors

## Abstract

Rifaximin is a broad-spectrum antibiotic that ameliorates symptomatology in inflammatory/functional gastrointestinal disorders. We assessed changes in gut commensal microbiota (GCM) and Toll-like receptors (TLRs) associated to rifaximin treatment in mice. Adult C57BL/6NCrl mice were treated (7/14 days) with rifaximin (50/150 mg/mouse/day, PO). Luminal and wall-adhered ceco-colonic GCM were characterized by fluorescent in situ hybridization (FISH) and microbial profiles determined by terminal restriction fragment length polymorphism (T-RFLP). Colonic expression of TLR2/3/4/5/7 and immune-related markers was assessed (RT-qPCR). Regardless the period of treatment or the dose, rifaximin did not alter total bacterial counts or bacterial biodiversity. Only a modest increase in *Bacteroides* spp. (150 mg/1-week treatment) was detected. In control conditions, only *Clostridium* spp. and *Bifidobacterium* spp. were found attached to the colonic epithelium. Rifaximin showed a tendency to favour their adherence after a 1-week, but not 2-week, treatment period. Minor up-regulation in TLRs expression was observed. Only the 50 mg dose for 1-week led to a significant increase (by 3-fold) in TLR-4 expression. No changes in the expression of immune-related markers were observed. Rifaximin, although its antibacterial properties, induces minor changes in luminal and wall-adhered GCM in healthy mice. Moreover, no modulation of TLRs or local immune systems was observed. These findings, in normal conditions, do not rule out a modulatory role of rifaximin in inflammatory and or dysbiotic states of the gut.

## 1. Introduction

Rifaximin is a semi-synthetic non-absorbable antibiotic derived from rifamycin and with a broad-spectrum activity against Gram-positive and Gram-negative microorganisms proposed to act on the gut microenvironment [1,2]. The main advantage of rifaximin over similar antibiotics is that it is virtually unabsorbable, which minimizes systemic exposure and adverse events in all patient populations [3]. Rifaximin showed to be effective for a variety of clinical uses and was initially approved for the treatment of traveler’s diarrhea caused by noninvasive strains of *E. coli* [1] and hepatic encephalopathy (due to its inhibition of ammonia-producing enteric bacteria and consequent reduction of circulating ammonia in patients with cirrhosis) [4]. Further clinical evidence led to the approval of the use of rifaximin for the treatment of diarrhea-predominant irritable bowel syndrome (IBS) [5]. Moreover, in the clinical practice, rifaximin is often prescribed for other gastrointestinal disorders, such as inflammatory bowel disease (IBD), small intestinal bacterial overgrowth (SIBO), and diverticular disease, because of its theoretical capability to modulate the intestinal microbiota [6].

IBS is a chronic, functional gastrointestinal disorder characterized by abdominal pain/discomfort, associated with altered bowel habits. The etiology of IBS is unknown and the pathophysiology is complex, heterogeneous, and not well understood. There is evidence for a number of underlying mechanisms, including altered intestinal barrier function, altered motility, visceral hypersensitivity and, possibly, a chronic, low-grade inflammatory or immunological response [7,8,9]. Moreover, interactions between environmental factors, such psychosocial stress and anxiety, led to the inclusion of brain-gut interactions in the etiology of the disease [7,8]. During the last years, gut microbiota has also been implicated in the pathogenesis of IBS. In this sense, some IBS patients have reduced gut microbial biodiversity [8] and acute enteric infections have been associated to the development of IBS, the so called post-infectious IBS [10]. Overall, the presence of dysbiosis, temporal or permanent, has been seen in more than 70% of patients with IBS [8], although great variability has been observed [11]. Nevertheless, the connection between dysbiosis and IBS is not completely understood, and a causal relationship has not been demonstrated [11,12]. It is known that gut commensal microbiota (GCM) contributes to maintenance of gastrointestinal homeostasis [13]. Therefore, modifying the GCM is a therapeutic approach of growing interest for IBS. In this context, rifaximin is used to treat SIBO and IBD [14], and, as mentioned above, was approved for the treatment of diarrhea-predominant IBS [15]. There is some clinical overlap between IBS and IBD, and some authors consider IBS and IBD at the two extremes of the same spectrum, being IBS a low-grade IBD without structural alterations [16]. In any case, dysbiosis seems to be a common finding in IBS and IBD.

Besides its antibacterial effects, pre-clinical evidence suggests that rifaximin might have anti-inflammatory activity, reducing mucosal inflammation and visceral hypersensitivity and restoring epithelial barrier function [17,18,19,20]. Whether or not these effects are secondary to its microbial actions or are direct, non-microbial-related, is still a matter of debate. Moreover, direct effects of rifaximin on intestinal epithelial cell physiology, associated with reductions in bacterial attachment and internalization and epithelial responses to inflammatory mediators, have also been suggested [19,21,22].

In this study, we assessed the effects of rifaximin on GCM in healthy mice. For this, we used fluorescence in situ hybridization (FISH) and a terminal restriction fragment length polymorphism (T-RFLP) analysis to determine rifaximin-induced changes in colonic luminal and wall adhered bacteria. To determine if changes in GCM might be associated to the local modulation of host immune-related responses, we also assessed (real-time qPCR) changes in the expression of toll-like receptors (TLR)-dependent host-bacterial interaction systems and immune-related markers.

## 2. Results

### 2.1. Effects of Rifaximin on Body Weight, Weight of Body Organs and Colonic Histology

Body weight was stable over the treatment period, without treatment-related significant changes (Table 1). At necropsy, the relative weight of the liver was slightly, but significantly reduced in rifaximin-treated animals, in similar proportion regardless the dose and the duration of treatment (*p* < 0.05 vs. respective vehicle-treated group). Relative weight of the spleen, thymus, and adrenal glands were similar across groups, (data not shown).

At necropsy, no macroscopic alterations were observed in the colon or cecum, irrespective of the experimental group considered. Similarly, cecal and colonic content had a normal consistency in all experimental groups. Colonic and cecal relative weight was similar across groups (Table 1). Consistent with these observations, no histological alterations were observed in the colon in rifaximin-treated animals, regardless the treatment time or dose. In all cases, total histological scores ranged between 0 and 2 (data not shown).

### 2.2. Effects of Rifaximin on Luminal GCM

**Figure 1:**
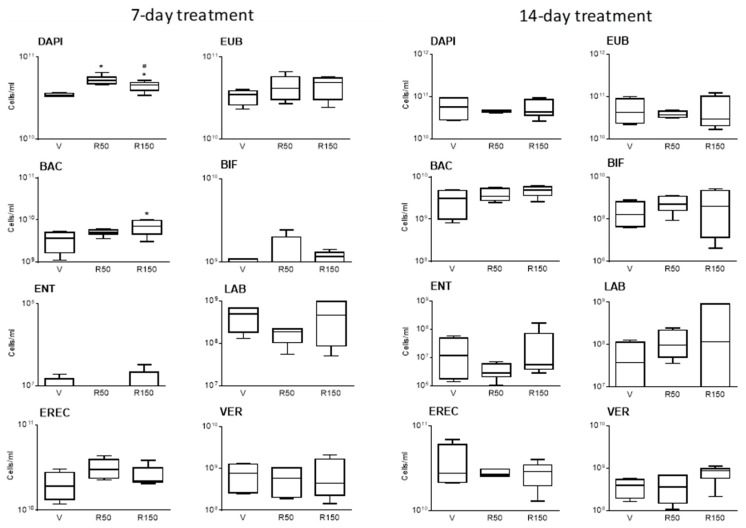
Colonic microbiota, as quantified by FISH after a 7-day or a 14-day treatment period with rifaximin. EUB: Total bacteria; BAC: *Bacteroides* spp.; ENT-D: Enterobacteria group; VER: Verrucobacteria group; BIF: *Bifidobacterium* spp.; LAB: *Lactobacillus*/*Enterococcus* spp.; EREC: *Clostridium* spp. cluster XIVa group. Data are media (interquartile range) ± SD, *n* = 4–6 per group. *: *p* < 0.05 vs. corresponding vehicle. #: *p* < 0.05 vs. rifaximin at 50 mg/kg. V: Vehicle; R50: rifaximin at 50 mg/kg; R150: rifaximin at 150 mg/kg.

**Figure 2:**
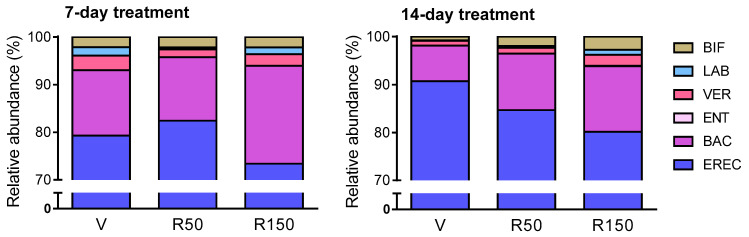
Relative distribution of the colonic microbiota, as quantified by FISH, in the different experimental groups. Data represent the relative abundance (percent) of the bacterial groups characterized [*Bacteroides* spp. (BAC), *Bifidobacterium* spp. (BIF), *Clostridium* spp. (EREC), Enterobacteria (ENT), *Lactobacillus* spp. (LAB), and Verrucobacteria (VER)]. Relative percent composition of the microbiota was calculated taking as 100% the total counts of the bacterial groups assessed. *n* = 4–6 per group. V: Vehicle; R50: rifaximin at 50 mg/kg; R150: rifaximin at 150 mg/kg.

In vehicle-treated animals, regardless the duration of treatment, total bacterial counts within the luminal content (EUB338-probe) oscillated between 2 × 10^10^ cells/mL and 1.5 × 10^11^ cells/mL (Figure 1); consistent with previous observations [23,24,25]. There was a good coincidence between total bacterial counts assessed by FISH (EUB338-probe) and DAPI staining (Figure 1). The most abundant bacterial group was *Clostridium* spp. (EREC482-probe), being within the order of 10^10^ cells/mL; followed by *Bacteroides* spp. (BAC303-probe) at 10^9^ cells/mL and Verrucobacteria (VER620-probe) at 10^8^ cells/mL. On the other hand, *Lactobacillus*/*Enterococcus* spp. and *Bifidobacterium* spp. were ranging between detection levels (10^6^ cells/mLL) and 10^8^–10^9^ cells/mL. Enterobacteria appeared below or at the threshold of detection levels (Figure 1 and Figure 2).

Total bacterial counts remained stable after the treatment with rifaximin, regardless the dose (50 or 150 mg/kg/day) or the duration of treatment considered (7-day or 14-day). Total counts oscillated between 2 × 10^10^ cells/mL and 7 × 10^10^ cells/mL and 1.5 × 10^10^ cells/mL and 1.5 × 10^11^ cells/mL for the 7-day and 14-day treatment period, respectively (Figure 1). Assessment of specific bacterial groups showed an increase in *Bacteroides* spp. (BAC303-probe) counts after a 7-day treatment period, but not after a 14-day period (Figure 1 and Figure 2). Although not significant, during the 14-day treatment, a dose-dependent reduction in the proportion of *Clostridium* spp. (EREC482-probe) was observed, at the expense of an increase in the relative abundance the other bacterial groups assessed, particularly *Bacteroides* spp. (BAC303-probe) (Figure 2).

**Figure 3:**
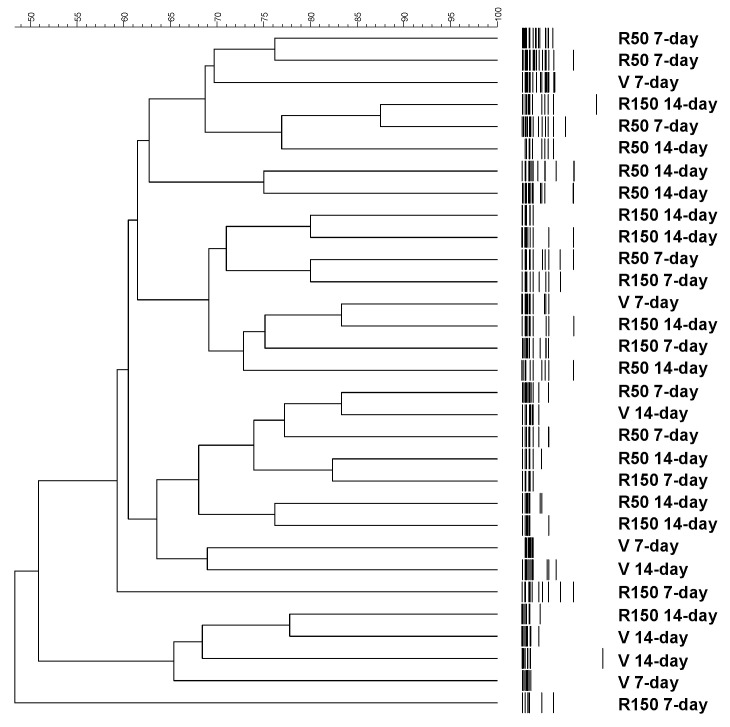
Ecological characterization of the luminal microbiota by T-RFLP analysis. Dendrogram showing the distribution of the different experimental groups according to the T-RFLP banding patterns obtained from the analysis of the ceco-colonic samples. Each line represents an animal identified by either R50 (rifaximin at 50 mg/kg), R150 (rifaximin at 150 mg/kg) or V (vehicle), followed by the experimental period (7-day or 14-day). The dendrogram distances represent percentage of similarity. The different experimental groups did not cluster together indicating the absence of treatment-related changes in the microbiota composition.

**Figure 4:**
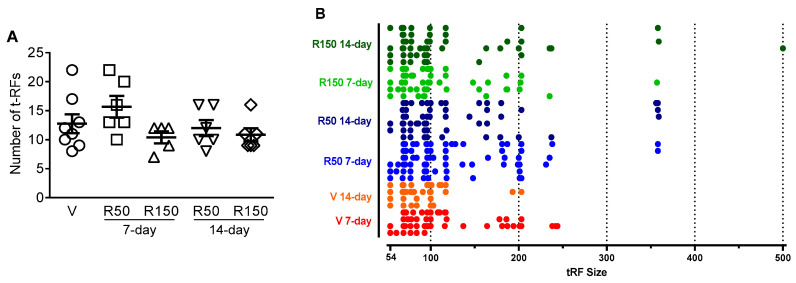
Ecological characterization of the luminal microbiota by T-RFLP analysis: Effects of rifaximin on biodiversity of the ceco-colonic microbiota. (**A**) Number of tRFs detected. Each symbol represents an individual animal; the horizontal lines with errors correspond to the mean ± SEM. For the sake of clarity, and since no differences were observed among them, vehicle-treated animals have been merged in a single group. V: Vehicle; R50: rifaximin at 50 mg/kg; R150: rifaximin at 150 mg/kg. (**B**) Distribution of the tRFs detected according to their size. Each line represents an individual animal and each column a tRF size. tRF distribution indicates a similar microbial biodiversity in all experimental groups, regardless the treatment applied. See also Table 2 for details regarding taxonomical classification of the tRFs detected.

The ecological characterization of the luminal microbiota was performed with a T-RFLP analysis. The dendrogram representation of the similarity indexes of the T-RFLP profiles of the ceco-colonic microbiota did not show a clustering of the different experimental groups (Figure 3). The number of t-RFs and their size distribution, taken as a measure of biodiversity, was similar across groups, regardless the dose (50 or 150 mg/kg/day) or the duration of treatment considered (7-day or 14-day) (*p* = 0.166; Figure 4).

Table 2 summarizes the main bacterial groups, as detected by the T-RFLP analysis, with differential presence in the six experimental groups (see also Figure 4B for distribution of the different tRF detected in function of their size). Overall, the T-RFLP analysis reveals high similarities in bacterial composition among the different experimental groups, without evident dose- or period of treatment-related changes in the diversity of the microbiota. Although similar bacterial groups were detected (according to the theoretical restriction 5′-fragment size), in many cases these groups could not be identified phylogenetically and were classified as “unidentified” or “uncultured bacterium”. According to the T-RFLP, tRFs with a size between 356 and 359 appeared in some rifaximin-treated animals, regardless the dose or the duration of treatment (Figure 4B). Although this might indicate some treatment-related effect, the low incidence observed (17–33%; Table 2) complicates its interpretation.

### 2.3. Effects of Rifaximin on Bacterial Adherence to the Colonic Wall

**Figure 5:**
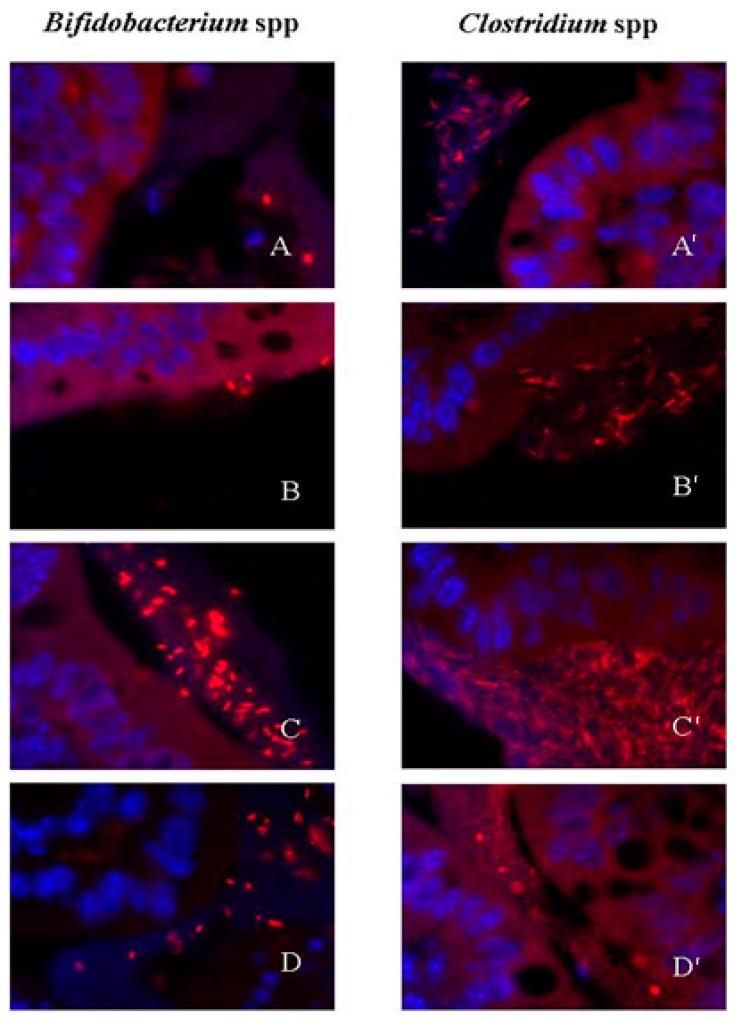
Bacterial wall adherence. Representative images (×100) showing bacterial adherence to the colonic epithelium for *Bifidobacterium* spp. and *Clostridium* spp. (**A**,**A'**) non-adhered bacteria within the intestinal lumen. (**B**,**B'**) adhered bacteria in a vehicle-treated mice (7-day), (**C**,**C'**) adhered bacteria in a rifaximin-treated mice (50 mg/kg, 7-day); (**D**,**D'**) adhered bacteria in a rifaximin-treated mice (150 mg/kg, 7-day).

**Figure 6:**
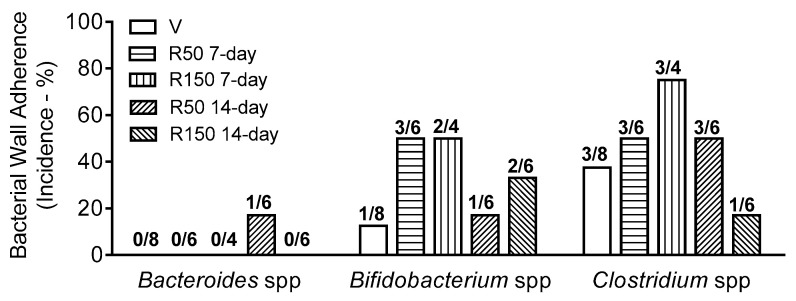
Incidence of bacterial wall adherence. Data represent the percentage of animals showing bacterial wall adherence. Numbers on top of columns represent the number of animals showing bacterial wall adherence over the total number of animals. For the sake of clarity, and since differences were not observed among them, vehicle-treated animals have been merged in a single group (*n* = 8). V: Vehicle; R50: rifaximin at 50 mg/kg; R150: rifaximin at 150 mg/kg. Because of technical problems, one of the samples from group R150 7-day was lost during its processing.

In vehicle-treated mice, *Bifidobacterium* spp. and *Clostridium* spp. were the only bacterial group attached to the colonic wall (epithelium). The overall incidence of attachment ranged from 12.5 to 37.5% (Figure 5 and Figure 6).

During antibiotic treatment for a 7-day period, there was a tendency to increase the incidence of bacterial wall adherence for *Clostridium* spp. (from an incidence of 37.5% in control conditions to 50 and 75% at 50 mg/kg and 150 mg/kg, respectively) and *Bifidobacterium* spp. (from an incidence of 12.5% in control conditions to 50% for both antibiotic-treated groups). However, this tendency disappeared in animals treated for a 14-day period (Figure 5 and Figure 6).

### 2.4. Effects of Rifaximin on Colonic Expression of TLRs and Immune-Related Markers

**Figure 7:**
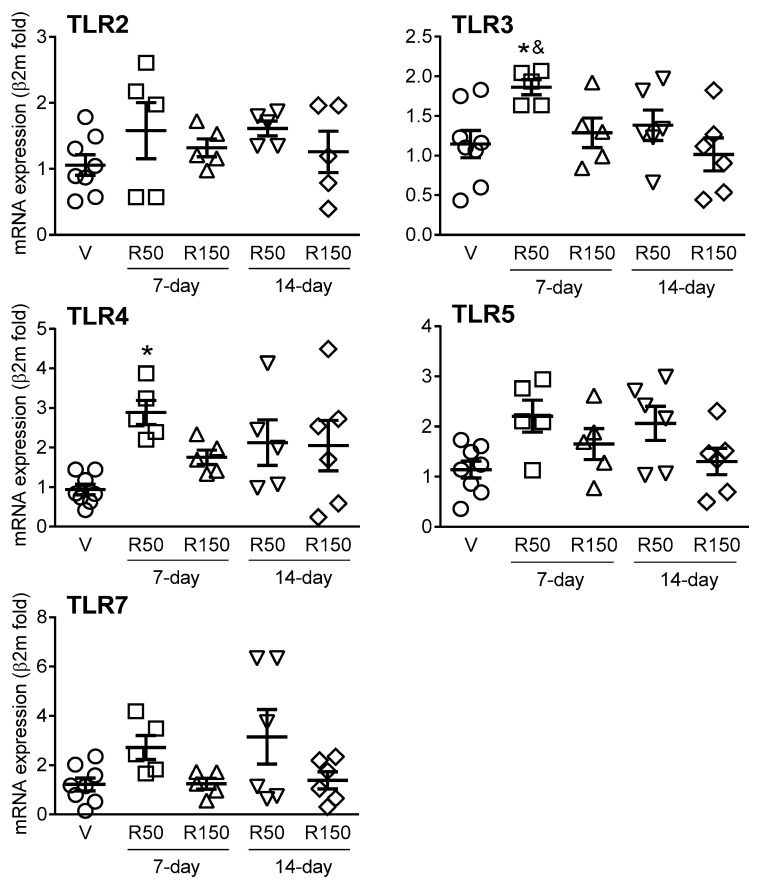
Effects of rifaximin on the colonic expression of TLRs. Each point represents an individual animal; the horizontal lines with errors correspond to the mean ± SEM. For the sake of clarity, and since no differences were observed among them, vehicle-treated animals have been merged in a single group (*n* = 8). *: *p* < 0.05 vs. V; &: *p* < 0.05 vs. R150 14-day. V: Vehicle; R50: rifaximin at 50 mg/kg; R150: rifaximin at 150 mg/kg.

Expression of TLR2, 3, 4, 5 and 7 was detected in all colonic samples. Overall, rifaximin induced minor changes in TLRs expression with only a moderate (2- to 3-fold), but significant, up-regulation observed for TLR3 and 4 for the 50 mg/kg dose during a 7-day period (Figure 7).

**Figure 8:**
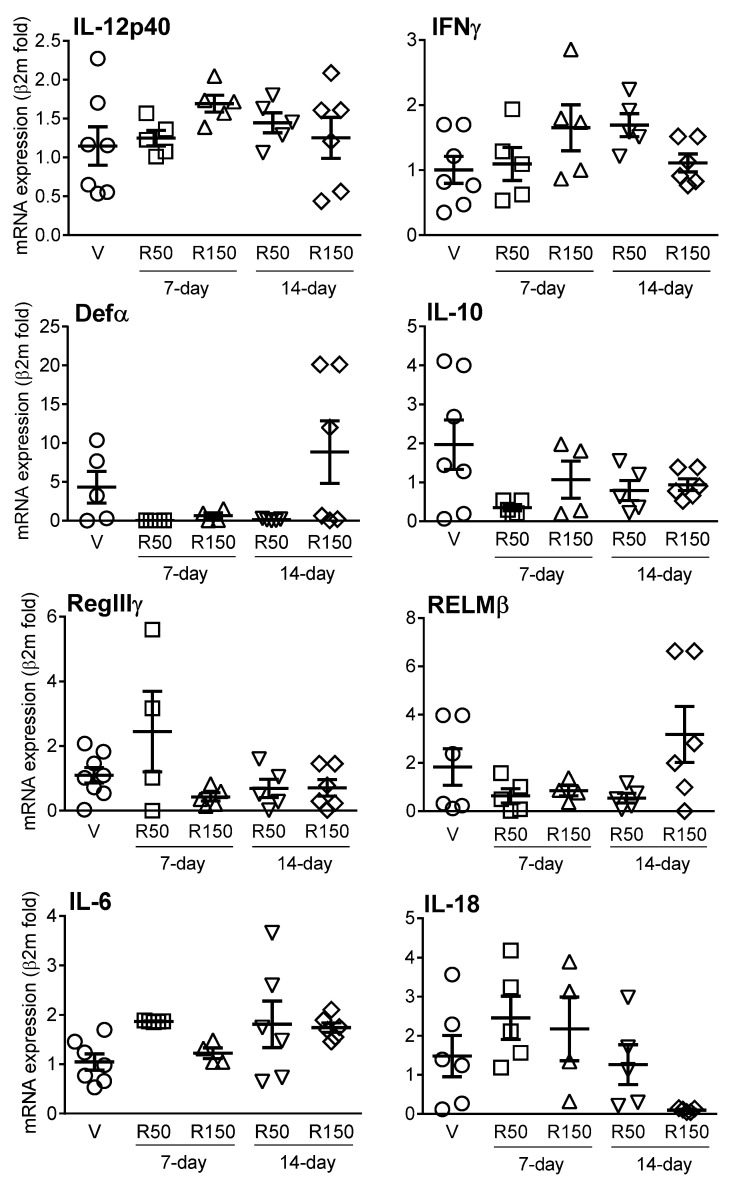
Effects of rifaximin on the colonic expression of immune-related markers. Each point represents an individual animal; the horizontal lines with errors correspond to the mean ± SEM. For the sake of clarity, and since no differences were observed among them, vehicle-treated animals have been merged in a single group (*n* = 8). V: Vehicle; R50: rifaximin at 50 mg/kg; R150: rifaximin at 150 mg/kg.

Expression of all immune-related markers assessed was detected in colonic tissues, although in some cases with relatively high variability. Regardless the dose and time of treatment, no changes were observed for pro-inflammatory cytokines (IL-6, IL-18, IFNγ and IL-12p40), anti-inflammatory cytokines (IL-10) or antimicrobial peptides (Defα24, RELMβ and RegIIIγ) (Figure 8).

## 3. Discussion

In the present study, we assessed the effects of the non-absorbable, wide spectrum antibiotic rifaximin on GCM in healthy mice. Overall, rifaximin did not alter the homeostatic state of the gastrointestinal tract expected in healthy animals. Results obtained show that rifaximin did not lead to major alterations in the microbial ecosystem of the gastrointestinal tract. Furthermore, rifaximin did not affect the local (colonic) expression of different immune-related markers associated to host-bacterial interactions.

The main focus of this study was on the ceco-colonic microbiota, since this region represents the largest proportion of the total gut microbiota and is also used to characterize dysbiosis in IBS and IBD [11]. Even though rifaximin has broad-spectrum activity against aerobic and anaerobic Gram-positive and Gram-negative bacteria, we show that during rifaximin treatment the GCM of standard, healthy mice was essentially not affected. Total bacterial counts in vehicle-treated mice were in the range previously described [24,26] and were not altered after a 7-day or a 14-day treatment with the antibiotic. In agreement with this, bacterial biodiversity, as assessed by a T-RFLP analysis, was not affected by rifaximin. This is consistent with previous studies showing no changes in total fecal bacterial counts after rifaximin treatment in patients with intestinal inflammation [2,27] or diarrheal disease [28] or in pre-clinical models [29]. However, these results contrast with data showing reductions in total fecal bacterial counts in rats after a 3-day treatment period at similar doses to those used here [30] or in ileal bacterial load in rats subjected to psychological stress [18,31]. These apparent discrepancies might reflect species-, treatment protocol- or disease state-related differences. Alternatively, a lack of changes might be related to a fast resilience-like response in which a quick adaptation and recovery of the microbiota might occur; since, comparatively, higher changes were observed after a 7-day vs. a 14-day treatment period.

A lack of effects of rifaximin inducing a clear dysbiosis in the present experimental conditions, against its expected antimicrobial effects and compared to other antibiotic treatments [23,32,33], might suggest a lack of efficacy of the treatment applied. However, the doses tested are in the range of those used in other reports showing biological activity [18,30,31,33]. Moreover, it does not seem related to a loss of activity of the antibiotic since in vitro testing using classical microbiological culture procedures showed efficacy against *S. aureus* and *E. coli* (data not shown).

Although without overall effects in total bacterial counts, rifaximin has been suggested to modulate the composition of the microbiota [2,19,27,34,35]. To assess this, we determined changes in specific ceco-colonic bacterial groups that are recognized as either beneficial (such as lactic acid bacteria like *Lactobacillus*/*Enterococcus* spp., and *Bifidobacterium* spp.) or harmful bacteria (such as some groups of *Clostridium* spp., *Bacteroides* spp., and Enterobacteria) [36]. The only bacterial group affected by rifaximin was *Bacteroides* spp., whose presence was favored by the antibiotic during the 7-day treatment, with a similar tendency observed during the 14-day treatment period. This is in agreement with recent studies in a murine model of ankylosing spondylitis in which rifaximin treatment increased the population of Bacteroidetes [34]. However, it contrasts with previous results showing exclusively an increase in *Bifidobacterium* spp. counts after a treatment with rifaximin [2,27]. Again, these contradictory results might be related to differences in the experimental conditions and/or reflect species related-differences (human vs. rodent). Composition of GCM is different in humans and rodents and differs significantly among rodent strains depending upon their breeder and their housing conditions [24,37]. For instance, rifaximin treatment also failed to affect microbiota in mice with a humanized microbiota [38], further emphasizing the importance of species-related aspects when assessing the microbiota.

The exact mechanisms by which rifaximin improves disease symptoms in IBD, IBS or diarrheic disease remain largely unknown [19]. In agreement with the limited effects of rifaximin in intestinal microbiota (in either normal or dysbiotic conditions) evidences suggest the existence of antibiotic-independent effects, likely modulating the local immune environment within the gastrointestinal tract as well as having direct effects on intestinal epithelial cells, modulating bacterial attachment and internalization and inhibiting intestinal bacterial translocation [19,21,22]. In this sense, changes in host–bacterial interaction, through the modulation of bacterial wall adherence, represent an attractive alternative mechanism of action, since only epithelium-attached bacteria are able to signal to the host leading to immune-related responses [19,39,40]. In our conditions, bacterial attachment to the colonic epithelium, as assessed by FISH of tissue samples from the colon, was only occasionally observed in control animals. In particular, only Clostridia and Bifidobacteria, including harmful and beneficial bacteria, respectively, were found attached to the colonic epithelium. Treatment with rifaximin did not affect this pattern, although a slight, and parallel, tendency to increase the incidence of attachment was observed for both bacterial groups during the 7-day treatment; with a progression towards control values for the 14-day treatment groups. The fact that rifaximin seems to promote the adherence of beneficial commensal bacteria at the same time than pathogenic microorganisms may contribute to its beneficial effects in the treatment of SIBO. In these conditions, the balance between negative and positive signals mediated through the interactions with harmful and beneficial bacteria, respectively, might inhibit dysbiosis-associated negative responses in the host, promoting the restoration of intestinal homeostasis, including a state of normobiosis.

Some evidences also suggest direct effects of rifaximin on intestinal epithelial cells, likely modulating local immune responses. To evaluate this possibility, we also assessed potential changes in the expression of local (colonic) immune-related markers during rifaximin treatment. In particular, we assessed the expression of TLR2, 3, 4, 5 and 7 and antimicrobial peptides (Defα24, RELMβ and RegIIIγ), according to their high expression within the gastrointestinal tract [40] and their implication in states of dysbiosis [41,42,43] as well as pro- (IL-6, INFɣ, IL-18, IL-12p40) and anti-inflammatory cytokines (IL-10), implicated in the development of colitis [44]. Overall, no significant changes were observed in the expression of cytokines, either pro- or anti-inflammatory, antimicrobial peptides or TLRs. In fact, only a moderate up-regulation was observed for TLR3 and TLR4 (50 mg, 7-day treatment). Few studies have addressed the effects of rifaximin on immune-related markers in control/healthy conditions. In this respect, no effects on inflammatory mediators after rifaximin treatment were observed in healthy animals treated with the antibiotic [17,30]. In states of altered gut function or chronic systemic inflammatory conditions (implicating also the gastrointestinal tract) both anti-inflammatory activity [34] and no effects on inflammation within the gut [17,38] have been reported for rifaximin. Similarly, Yang et al. (2019) [34] reported only a moderate down-regulation of intestinal TLR4 in a murine model of ankylosing spondylitis. Altogether, additional studies in control conditions (healthy animals) as well as in pathophysiological states involving the gastrointestinal tract are necessary to fully understand the direct, antibacterial-independent, effects of rifaximin on gastrointestinal immune responses. In our study, rifaximin was tested in standard, healthy animals, so we cannot exclude the possibility that the antibiotic might have a more pronounced effects in a disease-state model. This hypothesis warrants further follow-up studies. Moreover, additional studies confirming the current observation at the protein level (i.e., immunohistochemistry and/or Western blot) should also be performed.

As mentioned, several studies showed that antibiotic treatment in normal animals lead to a state dysbiosis concomitant to an immune activation, the induction of intestinal inflammation and the modulation of visceral sensory-related systems [23,32,33,45]. In our case, no evidence of immune activation or colonic alterations consistent with the development of an inflammatory-like state was observed upon macroscopic or microscopic examination of the colon. These differences are likely due to differences in the antibiotics used and their mechanism of action (including potential antimicrobial-independent effects, as discussed above). Nevertheless, additional, extended immune-related, including the flux of myeloid cells, as well as epithelial barrier function-related markers should be assessed in follow-up studies.

In summary, our results show that rifaximin, even though its antibacterial properties, induces very minor changes in GCM and bacterial wall adherence in normal mice, without changes in local immune-related markers. Although these observations, more noticeable effects of rifaximin on dysbiotic states, vs. a normal GCM, cannot be excluded. Therefore, further studies in dysbiotic animals should be performed to fully assess the effects and mechanism(s) of action of rifaximin within the gastrointestinal tract in order to fully understand the beneficial effects of the antibiotic in functional and inflammatory gastrointestinal disorders. These studies should include a broad assessment of immune-related markers and a deep characterization of the microbiota, including a metabolomics profiling, in order to detect minor, but functionally significant, changes within the microbiome.

## 4. Materials and Methods

### 4.1. Animals

Female C57BL/6NCrl mice (*n* = 31), 6 weeks old upon arrival, were obtained from Charles River Laboratories (Lyon, France). All animals were group-housed (2–4 animals per cage) under controlled temperature (20–22 °C) and photoperiod (12:12 h light-dark cycle) and had unrestricted access to standard mouse chow and tap water. Mice were allowed to acclimatize to these conditions for a 1-week period prior to any experimentation. The experiment was replicated twice at different time points. Females were used according to the higher prevalence of functional and inflammatory gastrointestinal disorders in women [46,47]. All procedures were approved by the Ethical Committee of the UniversitatAutònoma de Barcelona (protocols 1099 and 1101) and the Generalitat de Catalunya (protocols 5645 and 5646).

### 4.2. Antibiotic

Rifaximin [4-Deoxy-4′-methylpyrido(1′,2′-1,2)imidazo(5,4-c)rifamycin SV; reference: R9904, CAS Number 80621-81-4; Sigma-Aldrich, St. Louis, MO, USA] was suspended, under sonication, in sterile PBS at a final concentration of 50 mg/mL, then aliquoted and frozen (−20 °C) until use. Subsequent dilutions to obtain the desired concentrations were freshly made, on a daily basis, using sterile PBS. Sterile PBS was used as vehicle control.

### 4.3. Experimental Protocols

Upon arrival, mice were randomly divided into 6 experimental groups: (i) vehicle, 7-day treatment (*n* = 4); (ii) rifaximin, 7-day treatment at 50 mg/kg (*n* = 6); (iii) rifaximin, 7-day treatment at 150 mg/kg (*n* = 5); (iv) vehicle, 14-day treatment (*n* = 4); (v) rifaximin, 14-day treatment at 50 mg/kg (*n* = 6); (vi) rifaximin, 14 day-treatment at 150 mg/kg (*n* = 6). Animals were dosed by oral gavage (0.2 mL/mice/day) with either sterile PBS (vehicle) or the appropriate dose of rifaximin. All treatments were performed in the morning, between 8:00 and 10:00 AM; during 7 or 14 consecutive days depending upon the experimental group considered. At the time of dosing animals were also weighed. Animals were euthanized for samples extraction (see below) 24 h after the last treatment. Doses and duration of treatments were selected in agreement with previous reports addressing effects of rifaximin and antibiotic-induced dysbiosis in similar experimental conditions [18,22,30].

### 4.4. Samples Collection

Twenty-four hours after the last treatment animals were deeply anesthetized with isoflurane (Isoflo, Esteve, Barcelona, Spain) and euthanized by exsanguination through intracardiac puncture, followed by cervical dislocation. A laparotomy was performed and fecal samples from the ceco-colonic region obtained and frozen immediately in liquid nitrogen. All fecal samples were stored at −80 °C until analysis.

Thereafter, the cecum, colon, liver, spleen, thymus and adrenal glands were dissected and weighed. Tissue samples from the colon were fixed overnight in 4% paraformaldehyde or in Carnoy fixative (ethanol:chloroform:glacial acetic acid, 6:3:1, *v*:*v*:*v*) for histological studies. After fixing, tissues were paraffin embedded and 5 µm-tick sections obtained for either Hematoxylin-Eosin staining (4% paraformaldehyde-fixed tissues) or FISH (Carnoy-fixed tissues).

### 4.5. Histological Evaluation

For histological examination, hematoxylin-eosin-stained sections from the colon were obtained following standard procedures. Colonic histology was assessed following, with minor modifications, procedures previously described by us [23]. A histopathological score (ranging from 0, normal, to 12, maximal alterations) was assigned to each animal. Specifically, parameters scored included: epithelial structure (0: normal; 1: mild alterations of the epithelium; 2: local epithelial damage and/or fusion; 3: generalizedepithelial damage), structure of the crypts (0: normal; 1: mild alterations of the crypts; 2: local damage of the crypts; 3: generalized damage of the crypts), presence of edema (0: normal; 1: mild local edema in submucosa and/or lamina propria; 2: moderate diffuse edema in submucosa and/or lamina propria; 3: severe generalized edema in submucosa and/or lamina propria), presence of inflammatory infiltrate (0: normal; 1: mild localized infiltrate; 2: mild generalized infiltrate; 3: severe generalized infiltrate). Scoring was performed on coded slides by two independent researchers (MF and VM).

### 4.6. Bacterial Identification by Fluorescence in Situ Hybridization (FISH)

For FISH, previously characterized, bacterial-specific oligonucleotide probes consisted in a single strain DNA covalently linked with a Cy3 (carbocyanine) reactive fluorescent dye at the 5′ end (Biomers, Ulm/Donau, Germany and TibMolbiol, Mannheim, Germany) [23,24,32,48]. The bacterial groups characterized and the specific probes used are indicated in Table 3.

For the assessment of luminal bacteria by FISH previously published methods, with minor modifications, were followed [23,24,32,37,48]. Frozen fecal samples were thawed and 0.5 g of feces suspended in 4.5 mLof sterile and filtered PBS, including 2–4 glass beads (diameter 3 mm), and homogenized on a vortex mixer for 3 min. The fecal suspension was then centrifuged (1 min, 700 g, 4 °C) in order to remove large particles from the suspension. From the supernatant 0.5 μLwere collected and fixed in 1.5 mL freshly prepared 4% filtered paraformaldehyde solution. After overnight fixing at 4 °C, samples were aliquoted (6 portions of 200 μL and 2 portions of 400 μL) and stored at −20 °C until use. After thawing, fixed fecal samples were diluted in sterile and filtered PBS. Dilutions used were: 1600× and 800× for the EUB338-probe; 400× and 160× for the VER620-, EREC482- and BAC303-probes; 160× and 80× for the LAB158-probe; 40× and 80× for the BIF164- and ENT-D-probes. Ten-well slides with round-shaped wells (7 mm diameter) were used. In order to enhance adhesion of fecal bacteria to the slide, slides were pre-treated by soaking them in a gelatin-suspension 2% (5 mL: 0.1 g gelatin, 0.01 g KCr(SO_4_)·12H_2_O and miliQ water up to 100 mL) for 30 min and allowed to dry at room temperature. Subsequently, 5 μL of the proper dilution was pipetted in each separate well. After drying at room temperature, the slides were fixed for 10 min using 96% ethanol (*v*/*v*). Dilutions of the probe were made in TE Buffer (10 mMTris, 1 mM EDTA; Ambion, Austin, TX, USA) to a concentration of 50 ng/μL and then stored at −20 °C. Prior to use, the diluted probe solutions were further diluted in hybridization buffer (20 mM Tris–HCl, 0.9 M NaCl, 0.1% SDS, pH 7.2) to a concentration of 10 ng/μL and preheated at the corresponding temperature (see Table 3). Samples were hybridized in a dark moist chamber for 3 h by addition of the corresponding Cy3-labeled oligonucleotide probe. Treatments with formamide or lysozyme and hybridization temperatures were used as described, to achieve the optimal stringency (see Table 3 for details of hybridization conditions) [23,24,32,37,48,49]. Subsequently, the slides were rinsed in preheated washing buffer (20 mM Tris–HCl, 0.9 M NaCl, pH 7.2) for 30 min at the corresponding temperature (see Table 3). After briefly rinsing in milli-Q, the slides were air-dried and mounted with Vectashield-DAPI (Vector Laboratories, Peterborough, UK) on each well and a coverslip. The fluorescent stain 4′,6-diamidino-2-phenylindole (DAPI), which binds strongly to DNA, served as a control signal in all samples. Hybridized slides were viewed under oil immersion, using a Carl Zeiss Axioskop 40 FL epifluorescence microscope (filter for Cy3) equipped with a digital camera (Zeiss AxioCamMRm) for obtaining digital images (Zeiss AxioVision Release 4.8.1) (Carl Zeiss, Jena, Germany). For quantification of bacteria, 20 randomly selected fields were photographed, the number of hybridized cells counted using the CellC software [50], and the mean value obtained. All procedures were performed on coded slides, to avoid bias.

To assess wall-adhered bacteria, hybridization of tissue samples was also performed. Sections from Carnoy-fixed paraffin-embedded tissues were deparaffinized, rehydrated, post-fixed in 4% paraformaldehyde and washed. Hybridization conditions used were essentially as described above for luminal bacteria (see Table 3 for hybridization conditions), but in this case tissue samples were incubated for 16 h with the mix of hybridization buffer and the specific probe. In hybridized tissue samples, 20 randomly selected fields were photographed. Analysis of the images was performedmanually by two independent researchers (MF and VM) that observed the pictures and localizedhybridized bacteria within the mucus layer or attached to the epithelial surface. A coincidence between the two observers in bacterial location in at least 3 out of the 20 pictures observed (15%) was required to decide that there was bacterial attachment to the epithelium [23,24,32]. All procedures wereperformed on coded slides, to avoid bias.

### 4.7. Terminal Restriction Fragment Length Polymorphism

Terminal restriction fragment length polymorphism (T-RFLP) analysis of bacterial communities was performed following methods published elsewhere [37,51]. Briefly, a 1497-pb fragment of the 16S rDNA gene was amplified using a 6-carboxy-fluorescein-labeled forward and reverse primers (S-D-Bact-0008-a-S-20: 5′-6-FAM-AGAGTTTGATCMTGGCTCAG-3′; PH1552: 5′AAGGAGGTGATCCAGCCGCA-3′, respectively) against the first 20 bases of the 16S RNA sequence. Duplicate PCR were performed for each sample. Fluorescent-labeled PCR products were purified on QIAquick PCR purification kit columns (Qiagen, West Sussex, UK) and eluted in a final volume of 30μL of Milli-Q water. Then, the resultant PCR product was subjected to a restriction with HhaI (20,000 U/μL) (Biolabs Inc., Ipswich, MA, USA). Fluorescent-labeled terminal restriction fragments (TRF) were analyzed by capillary electrophoresis on an automatic sequence analyzer (ABI 3100 Genetic Analyzer, PE Biosystems, Warrington, UK) in Gene-Scan mode with a 25-U detection threshold. Determination of the TRF sizes in the range 50–700 bp were performed with the size standard GS-1000-ROX (PE Biosystems).

Data obtained consisted of size (base pairs) and peak area for each TRF. To standardize the DNA loaded on the capillary, the sum of all TRF peak areas in the pattern was used to normalize the peak detection threshold in each sample. Following the method described by Kitts [52], a new threshold value was obtained by multiplying a pattern’s relative DNA ratio (the ratio of total peak area in the pattern to the total area in the sample with the smallest total peak area) by 323 area units (the area of the smallest peak at the 25 detection threshold in the sample with the smallest total peak area). For each sample, peaks with a lower area were deleted from the data set. Thereafter, a new total area was obtained by the sum of all the remaining peak areas in each pattern.

Biodiversity (also known as richness) was considered as the number of peaks in each sample after standardization. For pair-wise comparisons of the profiles, a Dice coefficient was calculated, and dendrograms were constructed using the Fingerprinting II software (Informatix, Bio-Rad, Hercules, CA, USA) and an unweighted pair-group method with averaging algorithm. To deduce the potential bacterial composition of the samples, in silico restrictions for the major mouse gut bacteria with the primers and the enzyme used were obtained by using the analysis function TAP–T-RFLP from the Ribosomal Database Project II software. Results are presented as potential compatible bacterial species. Note also that direct attribution of species to individual peaks is not unequivocally possible unless fingerprinting is complemented with sequence analysis of clone libraries. Analysis of electropherograms was used for the visual comparison of compatible TRF with different bacteria for the different experimental groups.

### 4.8. Colonic Expression of TLRs and Immune-Related Markers Using Quantitative Real-Time PCR (RT-qPCR)

Total RNA was extracted from colonic tissue samples using Tri reagent with Ribopure Kit (Ambion/Applied biosystems, Foster City, CA, USA). RNA samples were converted into cDNA using a High Capacity cDNA Reverse Transcription Kit (Applied Biosystems). cDNA concentration was measured using NanoDrop (ND-1000 spectrophotometer, NanoDrop Technologies, Wilmington, DE, USA) and all the samples were diluted at 100 ng/µl with DEPC-Treated water (Ambion/Applied biosystems, Foster City, CA, USA). TaqMan gene expression assays for interleukin 6 (IL-6) (Mm00446190_m1), interferon gamma (IFNγ) Mm01168134_m1, interleukin-12 p40 (IL-12p40) (Mm00434174_m1), interleukin-10 (IL-10) (Mm00439614_m1), interleukin 18 (IL-18) (Mm00434225_m1), defensin alpha 24 (Defα24) (Mm04205950_gH), resistin-like molecule-β (RELMβ) (Mm00445845_m1), regenerating islet-derived protein 3 gamma (RegIIIγ) (Mm00441127_m1) and TLR2 (Mm00442346_m), TLR3 (Mm01207404_m1), TLR4 (Mm00445273_m1), TLR5 (Mm00546288_s1) and TLR7 (Mm00446590_m1) were used (Applied Biosystems). All samples, as well as the negative controls, were assayed in triplicates. β-2-microglobulin (β2m) (Mm00437762_m1) was used as endogenous control.

The PCR reaction mixture was incubated on the Bio-Rad CFX384 (Bio-Rad Laboratories). Bio-Rad CFX Manager 3.1 software was used to obtain the cycle threshold for each sample. All data was analyzed with the comparative Ct method (2-ΔΔCt) with the vehicle groups serving as calibrator [53].

### 4.9. Statistical Analysis

Data are expressed as mean ± SEM or media (interquartile range) ± SD, as indicated. A robust analysis (one iteration) was used to obtain mean ± SEM for RT-qPCR data. Data were analyzed by one-way analysis of variance (ANOVA), followed, when necessary, by a Student-Newman-Keuls multiple comparisons test. Data were considered statistically significant when *p* < 0.05. All statistical analysis were performed using GraphPad Prism 6 (GraphPad Software, La Jolla, CA, USA).

## Figures and Tables

**Table 1:** Body weight and relative weight of colon and cecum in the different experimental groups ^1^.

Treatment Duration	Treatment	*n*	Body Weight at Necropsy (g)	Body Weight (% Change from Day 0)	Colon Relative Weight (mg/cm)	Cecum Relative Weight (mg/g Body Weight)
7-day	Vehicle	4	17.6 ± 0.6	−4.6 ± 2.7	23.2 ± 1.7	21.5 ± 0.9
	Rifaximin (50 mg/kg)	6	17.8 ± 0.2	2.7 ± 2.4	21.8 ± 0.8	25.1 ± 2.3
	Rifaximin (150 mg/kg)	5	18.3 ± 0.2	−1.6 ± 1.5	21.3 ± 1.6	25.9 ± 0.9
14-day	Vehicle	4	17.4 ± 0.7	1.9 ± 2.2	21.7 ± 0.2	22.2 ± 0.8
	Rifaximin (50 mg/kg)	6	18.3 ± 0.3	−2.5 ± 2.1	22.9 ± 1.3	23.7 ± 1.9
	Rifaximin (150 mg/kg)	6	18.2 ± 0.2	2.1 ± 1.4	24.5 ± 0.7	21.2 ± 0.9

^1^ Data represent mean ± SEM of the number of animals indicated (*n*).

**Table 2:** Theoretical restriction 5′-fragment (tRF) size predicted for the major mouse gut bacteria and prevalence in the different experimental groups.

Compatible Bacterial Group	tRFSize	Frequency ^1^					
		V 7-day (*n* = 4)	V 14-day (*n* = 4)	R50 7-day (*n* = 6)	R50 14-day (*n* = 6)	R150 7-day (*n* = 5)	R150 14-day (*n* = 6)
Unidentified	54–55	3 (75)	4 (100)	4 (67)	3 (50)	3 (60)	5 (83)
*Bacillus* spp./*Lactococcus lactis* spp.	61–62	1 (25)	2 (50)	1 (17)	0 (0)	1 (20)	1 (17)
*Salinicoccusroseus*	63	0 (0)	0 (0)	1 (17)	0 (0)	0 (0)	0 (0)
*Burkholderia* spp./*Bordetella* spp./*Thiomonas* spp./ uncultured bacterium	67	0 (0)	0 (0)	0 (0)	0 (0)	1 (20)	1 (17)
*Mycobacterium* spp./ uncultured rumen bacterium	68	0 (0)	2 (50)	1 (17)	2 (33)	1 (20)	2 (33)
Uncultured rumen bacterium	69	4 (100)	4 (100)	5 (83)	5 (83)	4 (80)	6 (100)
Uncultured rumen bacterium/ *Leptotrichia* spp.	71	2 (50)	1 (25)	4 (67)	1 (17)	2 (40)	0 (0)
Uncultured rumen bacterium	72	1 (25)	1 (25)	1 (17)	3 (50)	2 (40)	1 (17)
*Photorhabdus* sp.	74–75	1 (25)	0 (0)	0 (0)	0 (0)	0 (0)	0 (0)
Uncultured bacterium	77	2 (50)	1 (25)	3 (50)	3 (50)	1 (20)	5 (83)
Uncultured rumen bacterium/ naphthalene-utilizing bacterium	78	1 (25)	3 (75)	1 (17)	1 (17)	1 (20)	3 (50)
Uncultured bacterium	80	1 (25)	0 (0)	2 (33)	0 (0)	1 (20)	0 (0)
*Sphingomonas* spp./ uncultured bacterium	81	0 (0)	1 (25)	0 (0)	0 (0)	0 (0)	0 (0)
Uncultured bacterium	84	3 (75)	2 (50)	2 (33)	1 (17)	0 (0)	1 (17)
*Desulfovibriodefluvii*/ *Roseiflexus* spp.	86–87	2 (50)	1 (25)	1 (17)	2 (33)	1 (20)	5 (83)
*Flavobacterium psychrophilum*	88–89	0 (0)	0 (0)	0 (0)	0 (0)	0 (0)	1 (17)
*Flavobacterium johnsoniae*	90	1 (25)	0 (0)	0 (0)	1 (17)	0 (0)	0 (0)
*Anaeromyxobacter dehalogenans*/ uncultured bacterium	91	0 (0)	1 (25)	0 (0)	1 (17)	2 (40)	0 (0)
*Geobacter* spp./uncultured Bacteroidetes/ *Cytophaga* spp./ *Algoriphagus* spp.	92	0 (0)	0 (0)	1 (17)	0 (0)	0 (0)	0 (0)
*Flavobacteriaceae bacterium*/uncultured rumen bacterium/ *Desulfovibrio* spp.	93–94	2 (50)	0 (0)	3 (50)	4 (67)	2 (40)	5 (83)
*Desulfovibrio profundus*/ uncultured bacterium	95	2 (50)	0 (0)	3 (50)	1 (17)	1 (20)	0 (0)
Uncultured bacterium	96	1 (25)	1 (25)	3 (50)	4 (67)	2 (40)	4 (67)
*Desulfococcus oleovorans*/ *Desulfomonile limimaris*/ Helicobacter pylori	97–98	1 (25)	0 (0)	4 (67)	4 (67)	2 (40)	4 (67)
Helicobacter pylori/ uncultured rumen bacterium	99		2 (50)	1 (17)	2 (33)	1 (20)	5 (83)
*Bacteroides* spp./ uncultured rumen bacterium	100	3 (75)	3 (75)	2 (33)	1 (17)	2 (40)	0 (0)
*Bacteroides fragilis*/ uncultured rumen bacterium/ *Prevotella ruminicola*	101–102	1 (25)	2 (50)	0 (0)	0 (0)	0 (0)	0 (0)
Uncultured rumen bacterium	103–104	1 (25)	1 (25)	1 (17)	0 (0)	0 (0)	0 (0)
Uncultured bacterium	105	0 (0)	0 (0)	1 (17)	0 (0)	0 (0)	0 (0)
*Desulfitobacterium hafniense*	107–108	1 (25)	0 (0)	0 (0)	0 (0)	0 (0)	0 (0)
*Thiobacillus* spp.	110–111	1 (25)	2 (50)	0 (0)	0 (0)	1 (20)	0 (0)
Uncultured bacterium	112	0 (0)	1 (25)	0 (0)	1 (17)	0 (0)	0 (0)
Unidentified	113	0 (0)	0 (0)	0 (0)	1 (17)	0 (0)	1 (17)
Uncultured bacterium	116	2 (50)	0 (0)	0 (0)	0 (0)	0 (0)	0 (0)
Unidentified	117	1 (25)	2 (50)	4 (67)	3 (50)	2 (40)	5 (83)
*Desulfitobacterium hafniense*	118	1 (25)	0 (0)	2 (33)	1 (17)	1 (20)	0 (0)
Unidentified	123–124	0 (0)	0 (0)	1 (17)	0 (0)	0 (0)	0 (0)
Unidentified	127–129	0 (0)	0 (0)	1 (17)	0 (0)	0 (0)	0 (0)
Unidentified	136	0 (0)	0 (0)	1 (17)	0 (0)	0 (0)	0 (0)
Uncultured rumen bacterium	137	1 (25)	0 (0)	1 (17)	0 (0)	0 (0)	0 (0)
*Microbacterium* spp.	144–145	0 (0)	0 (0)	0 (0)	1 (17)	0 (0)	0 (0)
*Leucobacter* spp./ *Janibacter* spp.	147	0 (0)	0 (0)	1 (17)	0 (0)	0 (0)	0 (0)
Unidentified	148–149	0 (0)	0 (0)	2 (33)	0 (0)	1 (20)	0 (0)
*Pseudomonas aeruginosa*	155	0 (0)	0 (0)	0 (0)	1 (17)	1 (20)	1 (17)
Unidentified	156	1 (25)	0 (0)	0 (0)	1 (17)	0 (0)	0 (0)
Unidentified	163	0 (0)	0 (0)	0 (0)	1 (17)	1 (20)	1 (17)
*Synechococcus* spp.	164	1 (25)	0 (0)	0 (0)	3 (50)	0 (0)	0 (0)
Unidentified	165–167	0 (0)	0 (0)	3 (50)	0 (0)	1 (20)	0 (0)
Uncultured bacterium	178	1 (25)	0 (0)	0 (0)	0 (0)	0 (0)	0 (0)
Uncultured rumen bacterium	179	0 (0)	0 (0)	0 (0)	1 (17)	0 (0)	1 (17)
Uncultured rumen bacterium	180	0 (0)	0 (0)	0 (0)	2 (33)	0 (0)	0 (0)
Uncultured rumen bacterium	181–182	1 (25)	0 (0)	2 (33)	2 (33)	0 (0)	0 (0)
Uncultured rumen bacterium	183	0 (0)	0 (0)	1 (17)	0 (0)	0 (0)	0 (0)
Uncultured rumen bacterium	184–185	1 (25)	0 (0)	2 (33)	0 (0)	1 (20)	0 (0)
*Listeria monocytogenes*	186	1 (25)	0 (0)	0 (0)	0 (0)	1 (20)	0 (0)
Uncultured rumen bacterium	187	1 (25)	0 (0)	1 (17)	0 (0)	1 (20)	1 (17)
Uncultured rumen bacterium	193	0 (0)	1 (25)	0 (0)	0 (0)	0 (0)	0 (0)
*Psychrobacter* spp./ uncultured bacterium/ *Francisella* spp.	194–195	1 (25)	0 (0)	0 (0)	0 (0)	0 (0)	0 (0)
Unidentified	199	0 (0)	0 (0)	1 (17)	0 (0)	0 (0)	0 (0)
*Bacillus* spp.	200	0 (0)	0 (0)	1 (17)	1 (17)	0 (0)	0 (0)
*Clostridium rectum*/ uncultured bacterium/ *Mycobacterium* spp.	201	1 (25)	0 (0)	3 (50)	0 (0)	1 (20)	1 (17)
*Fervidobacterium* spp./ *Dehalococcoides* spp./ uncultured bacterium	202	0 (0)	0 (0)	0 (0)	0 (0)	1 (20)	0 (0)
Uncultured rumen bacterium	203–204	2 (50)	1 (25)	3 (50)	1 (17)	1 (20)	5 (83)
*Clostridium* spp.	231–232	0 (0)	0 (0)	1 (17)	0 (0)	0 (0)	0 (0)
*Clostridium perfringens*	234–235	0 (0)	0 (0)	1 (17)	0 (0)	1 (20)	1 (17)
*Clostridium botulinum*	237	0 (0)	0 (0)	0 (0)	1 (17)	0 (0)	1 (17)
*Bacillus subtilis* subsp. Subtilis	238	1 (25)	0 (0)	1 (17)	0 (0)	0 (0)	0 (0)
*Bacillus subtilis* subsp. subtilis/*Bacillus licheniformis*/*Bacillus* spp.	241–242	1 (25)	0 (0)	0 (0)	0 (0)	0 (0)	0 (0)
*Geobacillus stearothermophilus*/*Paenibacillus* spp.	244	1 (25)	0 (0)	0 (0)	0 (0)	0 (0)	0 (0)
*Microbispora* spp./ *Pseudonocardia compacta*/*Nonomuraea bangladeshensis*/*Kineosporia aurantiaca*	356	0 (0)	0 (0)	0 (0)	1 (17)	0 (0)	0 (0)
*Microbispora* spp./ *Herbidospora* spp.	357	0 (0)	0 (0)	0 (0)	0 (0)	1 (20)	0 (0)
*Kribbella* spp./ *Actinomadura* spp./ *Pseudonocardia* spp./ *Anaplasma marginale*	358	0 (0)	0 (0)	2 (33)	2 (33)	0 (0)	1 (17)
*Arthrobacter* spp.	359	0 (0)	0 (0)	0 (0)	1 (17)	0 (0)	1 (17)
Uncultured bacterium	500	0 (0)	0 (0)	0 (0)	0 (0)	0 (0)	1 (17)

^1^ Data represent the number of animals within each group presenting the bacterial group predicted by the corresponding tRF size and the incidence, in percentage (between brackets).

**Table 3:** Probes used for FISH and hybridization conditions.

Probe	Primer (5′→3′)	Target	Hybridization Conditions		
			Temp (°C)	Formamide	Lysozyme
EUB338	GCTGCCTCCCGTAGGAGT	All bacteria	50		
NON338	ACATCCTACGGGAGGC	Non bacteria (negative control)	50		
BAC303	CCAATGTGGGGGACCTT	*Bacteroides* spp.	48		
EREC482	GCTTCTTAGTCAGGTACCG	Clostridiumcoccoides cluster XIVa	50		
LAB158	GGTATTAGCACCTGTTTCCA	*Lactobacillus*- *Enterococcus* spp.	50		90 min, 37 °C
ENT-D	TGCTCTCGCGAGGTCGCTTCTCTT	Enterobacteria	50		
VER620	ATGTGCCGTCCGCGGGTT	Verrucobacteria	50	30%	
BIF164	CATCCGGCATTACCACCC	*Bifidobacterium* spp.	50		

## Data Availability

All data presented in this study are available on request from the corresponding author (vicente.martinez@uab.es).
